# Pathogenesis of Primary Aldosteronism: Impact on Clinical Outcome

**DOI:** 10.3389/fendo.2022.927669

**Published:** 2022-06-23

**Authors:** Lucas S. Santana, Augusto G. Guimaraes, Madson Q. Almeida

**Affiliations:** ^1^ Unidade de Adrenal, Laboratório de Hormônios e Genética Molecular Laboratório de Investigação Médica 42 (LIM/42), Serviço de Endocrinologia e Metabologia, Hospital das Clínicas, Faculdade de Medicina da Universidade de São Paulo, São Paulo, Brazil; ^2^ Divisão de Oncologia Endócrina, Instituto do Câncer do Estado de São Paulo (ICESP), Faculdade de Medicina da Universidade de São Paulo, São Paulo, Brazil

**Keywords:** primary aldosteronism, aldosterone, aldosterone synthase, genetics, outcome

## Abstract

Primary aldosteronism (PA) is the most common form of secondary arterial hypertension, with a prevalence of approximately 20% in patients with resistant hypertension. In the last decade, somatic pathogenic variants in *KCNJ5*, *CACNA1D, ATP1A1* and *ATP2B3* genes, which are involved in maintaining intracellular ionic homeostasis and cell membrane potential, were described in aldosterone-producing adenomas (aldosteronomas). All variants in these genes lead to the activation of calcium signaling, the major trigger for aldosterone production. Genetic causes of familial hyperaldosteronism have been expanded through the report of germline pathogenic variants in *KCNJ5, CACNA1H* and *CLCN2* genes. Moreover, *PDE2A* and *PDE3B* variants were associated with bilateral PA and increased the spectrum of genetic etiologies of PA. Of great importance, the genetic investigation of adrenal lesions guided by the CYP11B2 staining strongly changed the landscape of somatic genetic findings of PA. Furthermore, CYP11B2 staining allowed the better characterization of the aldosterone-producing adrenal lesions in unilateral PA. Aldosterone production may occur from multiple sources, such as solitary aldosteronoma or aldosterone-producing nodule (classical histopathology) or clusters of autonomous aldosterone-producing cells without apparent neoplasia denominated aldosterone-producing micronodules (non-classical histopathology). Interestingly, *KCNJ5* mutational status and classical histopathology of unilateral PA (aldosteronoma) have emerged as relevant predictors of clinical and biochemical outcome, respectively. In this review, we summarize the most recent advances in the pathogenesis of PA and discuss their impact on clinical outcome.

## Introduction

Arterial hypertension (AH) represents one of the main risk factors for premature death, affecting about 10 to 40% of the world population (1, 2). Primary aldosteronism (PA) is the most frequent cause of endocrine AH, with a prevalence of around 4% and 10% in hypertensive patients treated in primary and tertiary care services, respectively, reaching around 20% of patients with resistant AH (3–6).

PA is characterized by autonomous production of aldosterone, independent of the renin-angiotensin system. As a consequence, sodium retention, plasma renin suppression, blood pressure (BP) elevation and K^+^ excretion increase occur, with consequent cardiovascular damage (7). The latter is due to the fact that excess of aldosterone exerts its deleterious cardiovascular effects independent of blood pressure levels, resulting in higher cardiovascular morbidity and mortality in patients with PA when compared with patients with essential AH (8, 9).

Aldosterone is a mineralocorticoid hormone, which is synthesized by the zona glomerulosa (ZG) of the adrenal cortex. Its play a major role in electrolyte regulation through sodium and water renal reabsorption (10, 11). Aldosterone is synthetized from cholesterol and its biosynthesis is under the control of two principal factors: angiotensin II (Ang II) and extracellular potassium concentration (K^+^) (10).

Stimulation of ZG cells by Ang II or an increase in plasma K^+^ concentration leads to cell membrane depolarization and increase in intracellular Ca^2+^, by opening of voltage-gated Ca^2+^ channels and inositol triphosphate-dependent Ca^2+^ release from the endoplasmic reticulum. The increase of intracellular Ca^2+^ leads to activation of a phosphorylation cascade that positively regulate aldosterone synthesis and cell proliferation, specifically by increasing the *CYP11B2* gene transcription (10, 12, 13).

Effects of aldosterone are mediated through the mineralocorticoid receptor (MR), a hormone dependent transcription factor that is expressed in non-epithelial tissues, such as the heart and vessels, and in epithelial tissues such as the salivary glands and kidney distal tubule, where aldosterone regulates sodium/water reabsorption and potassium excretion (10).

The main causes of PA are bilateral cortical adrenal hyperplasia (idiopathic hyperaldosteronism) and aldosteronomas (14). Idiopathic hyperaldosteronism is caused by bilateral nodular hyperplasia originating from the cortical zona glomerulosa, whereas aldosteronomas are aldosterone-producing adenomas usually measuring between 1-3 cm (but can even measure less than 1 cm). Each of these accounts for about 50-60% and 40-50% of PA cases, respectively (7, 14).

The two major causes of PA account for more than 95% of cases, with approximately 5% of bilateral hyperplasia occurring in a familial context. Thus, bilateral hyperplasia remains without a defined genetic etiology in most cases. Although somatic allelic variants are identified in about 90% of aldosteronomas, few advances have been made in the genetic elucidation of bilateral PA (10, 15).

**Figure 1:**
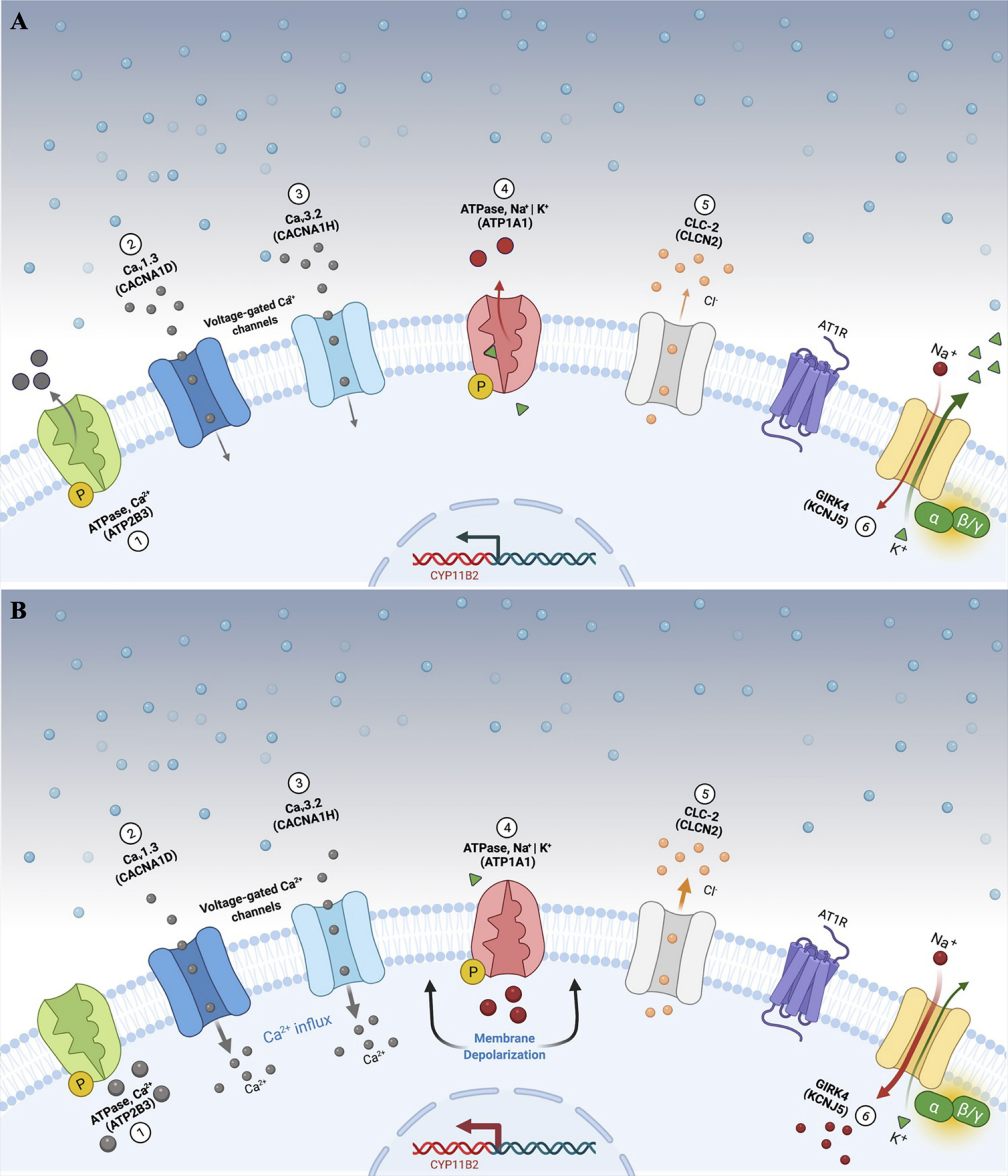
Aldosterone secretion in adrenal zona glomerulosa cells under physiological **(A)** and pathological **(B)** conditions. **(A)** Adrenal glomerulosa cell resting. The normal resting potential of zona glomerulosa cells is hyperpolarized (highly negative membrane potential). Activation of the angiotensin receptor (AT1R) by angiotensin II or extracellular hyperkalemia results in membrane depolarization and calcium influx *via* activated voltage-gated calcium channels. Calcium influx activates signaling to increase expression of aldosterone synthase (*CYP11B2*) and ultimately aldosterone production. **(B)** Genetic alterations leading to cell membrane depolarization, intracellular ionic modification, and autonomous aldosterone secretion in PA. Pathogenic variants in the KCNJ5 gene (G-protein-activated inward rectifier potassium channel GIRK4) [6] promote loss of channel K^+^ selectivity and increase permeability to Na^+^, leading to membrane depolarization and calcium influx via voltage-gated calcium channels. Similarly, impaired ATPase, Ca^2+^ (ATP2B3) [1]; Ca_v_1.3 (CACNA1D) [2]; Ca_v_3.2 (CACNA1H) [3]; ATPase, Na^+^ | K^+^ (ATP1A1) [4], and CLC-2 (CLCN2) [5] function results in cell membrane depolarization, calcium influx and autonomous aldosterone secretion. PA, primary aldosteronism.

Several genes that encode ion channels that modulate zona glomerulosa cell depolarization and aldosterone synthesis pathways have already been associated with the pathogenesis of PA (**Figure 1**), differing in prevalence among aldosteronomas and familial PA cohorts (12). The aim of this review is to discuss the most recent discoveries about the PA pathogenesis, as well as the clinical and prognostic impact of the genetic characterization of this very prevalent disorder, associated with a high cardiovascular morbidity.

## Diagnosis and Clinical Management

According to the American Endocrine Society (The Endocrine Society), the following scenarios are indicated for PA screening in hypertensive patients: I) AH and hypokalemia (spontaneous or induced by diuretic therapy); II) AH and adrenal incidentaloma; III) Blood pressure >150x100 mmHg on three different occasions; IV) AH not controlled (≥140/90 mmHg) on three or more antihypertensive drugs (resistant AH); V) controlled AH (<140x90 mmHg) on four antihypertensive drugs (resistant AH); VI) AH associated with obstructive sleep apnea; VII) AH and family history of AH or cerebrovascular disease of the young (<40 years); VIII) AH in first-degree relatives of patients with PH (7).

PA screening should be performed with plasma aldosterone (A) and renin (R) measurements, with hypokalemia correction before the test. To avoid false negative results, diuretics and spironolactone should be withheld for at least 4 to 6 weeks before the test. Aldosterone concentration >10 ng/dL and an A/PRA ratio (plasma renin activity) ≥ 30 ng/dL/ng/mL/h or A/R ≥2.0 confer a sensitivity and specificity greater than 90% for PH diagnosis (7, 16, 17). It should be emphasized that A/PRA or A/R ratio should be calculated only for patients with suppressed or very low renin levels.

After laboratorial PA confirmation, patients should undergo adrenal computed tomography (CT) for etiologic characterization and exclusion of adrenal cortical carcinoma. Adrenal CT has limited accuracy (around 60-70%), especially for detection of small (<1 cm) aldosteronomas (7) or for patients with bilateral nodules (to differentiate non-functioning or cortisol producing-adenomas). Therefore, adrenal vein sampling (AVS) is indicated for the majority of patients with PA for the proper characterization of the lateralization of aldosterone production (7, 16). Although AVS is the gold standard approach to define aldosterone lateralization, it should be carried out only in centers with expertise for this procedure and by a very experienced interventional radiologist. In addition, AVS should be considered only if laparoscopic surgery is a treatment option. A detailed description of PA work-up investigation is beyond the scope of this review.

Treatment of unilateral PA consists of laparoscopic adrenalectomy. The bilateral hyperplasia is treated with a mineralocorticoid antagonist (spironolactone or eplerenone). Both approaches are associated with reduced cardiovascular morbidity caused by excess of aldosterone (18, 19). The reduction of cardiovascular risk after medical treatment for PA is associated with normalization of renin levels (PRA >1 ng/mL/h) (20).

## Familial Hyperaldosteronism

Familial hyperaldosteronism (FH) is rare, but likely a highly underdiagnosed entity due to lack of routine screening (**Table 1**). Therefore, there is a lack of prevalence data for most of pathogenic variants listed in **Table 1**. The first report of FH occurred in 1966 (30), with subsequent characterization of its molecular etiology (21). This autosomal dominant form of PH was caused by a chimeric gene consisting of the 11β-hydroxylase promoter (*CYP11B1*) and aldosterone synthase (*CYP11B2*) coding region, resulting from a non-homologous pairing during crossing-over. Therefore, aldosterone synthesis becomes now regulated by adrenocorticotropic hormone (ACTH) instead of Ang II (21). This presentation of familial PH was then termed FH type 1 (OMIM #103900), or glucocorticoid-suppressible hyperaldosteronism.

**Table 1:** Genetic causes of familial hyperaldosteronism.

Gene (OMIM)	First Report	Prevalence	Discovery Approach	Features
*CYP11B1* (*610613)	1992 (21)	–	Kindred | Linkage	Chimeric *CYP11B1*/*CYP11B2* gene; PA remission by glucocorticoid treatment; variable age at onset (childhood to adult) (21, 22)
*KCNJ5* (*600734)	2011 (23)	7% (FH) | 0.3% (PA) (24)	Cohort | Exome	Early onset (first decade of life); medication-resistant hypertension; hypokalemia; bilateral adrenal macronodular hyperplasia (24)
*CACNA1D* (*114206)	2013 (25)	–	Cohort | Exome	Early onset (at birth/first decade of life); seizures; neurologic abnormalities; cardiomyopathy (25)
*CACNA1H* (*607904)	2015 (26)	–	Cohort | Exome	Early onset (usually in the first decade of life); incomplete penetrance (26, 27)
*CLCN2* (*600570)	2018 (28)	–	Cohort | Exome	Early onset (usually before 20 years of age); incomplete penetrance; variable expressivity; favorable response to spironolactone (29)

FH, familial hyperaldosteronism; PA, primary aldosteronism.

A diagnosis of FH 1 is highly suggestive if aldosterone suppression (<4 ng/dL) occurs after a dexamethasone suppression test (0.5 mg each 6h for 48h). However, the FH 1 diagnosis should be confirmed by the presence of the chimeric gene in a long range PCR (31). The treatment of FH 1 consists of low dose dexamethasone administration in adults (0.125–0.25mg/d) to suppress ACTH and block aldosterone synthesis (32, 33). If additional blood pressure control is required, a mineralocorticoid antagonist can be added.

The molecular pathogenesis of Type 2 FH (OMIM #605635) consists of gain-of-function heterozygous germline variants in the *CLCN2* gene (**Table 1**). Type 2 FH is characterized by autosomal dominant inheritance, incomplete penetrance and a family history of aldosteronoma or bilateral PA (34, 35). *CLCN2* was mapped as a FH gene in 2018 and encodes an inwardly rectifying chloride channel (ClC-2), a member of the CLC voltage-gated Cl^–^ channels family which is expressed in the cortical zona glomerulosa (28, 29).

So far, 6 missense pathogenic variants in *CLCN2* have been reported in the literature associated with FH 2 (**Table 2**) (48). The presence of these alleles causes an increase in Cl^-^ conductance through the channel, leading to a continuous depolarization of the plasma membrane, resulting in an increase of *CYP11B2* expression and consequent stimulus for aldosterone synthesis (**Figure 1B**) (28, 29).

**Table 2:** Germline allelic variants identified in probands with (familial) primary hyperaldosteronism/(early onset) hypertension.

Gene	Nucleotide change^1^	Aminoacid change^1^	Region	Families	ACMG^2,3^	Reference (first report)
**(familial) Primary Hyperaldosteronism**						
*CLCN2*	c.65T>A	p.(Met22Lys)	Exon 2	1	VUS-Cool	Scholl et al., 2018 (29)
	c.71G>A	p.(Gly24Asp)		1	VUS-Hot	Fernandes-Rosa et al., 2018 (28)
	c.76T>A	p.(Tyr26Asn)		1	VUS-Cool	Scholl et al., 2018 (29)
	c.515G>A	p.(Arg172Gln)	Exon 5	8	P	
	c.1084A>T	p.(Lys362*)	Exon 10	1	VUS-Tepid	
	c.2593A>C	p.(Ser865Arg)	Exon 24	1	VUS-Cool	
*KCNJ5*	c.155G>A	p.(Arg52His)	Exon 2	2	VUS-Tepid	Murthy et al., 2014 (36)
	c.433G>C	p.(Glu145Gln)		3	LP	Monticone et al., 2015 (37)
	c.452G>A	p.(Gly151Glu)		3	P	Mulatero et al., 2012 (38)
	c.451G>A	p.(Gly151Arg)		2	P	Scholl et al., 2012 (39)
	c.455A>G	p.(Tyr152Cys)		1	VUS-Hot	Monticone et al., 2013 (40)
	c.470T>G	p.(Ile157Ser)		1	VUS-Warm	Charmandari et al., 2012 (41)
	c.472A>G	p.(Thr158Ala)		3	LP	Choi et al., 2011 (23)
	c.736G>A	p.(Glu246Lys)		1	VUS-Warm	Murthy et al., 2014 (36)
	c.446_448del	p.(Thr149del)		1	VUS-Hot	Pons Fernández et al., 2019 (42)
*CACNA1H*	c.587C>T	p.(Ser196Leu)	Exon 5	1	VUS-Tepid	Daniil et al., 2016 (27)
	c.2669G>A	p.(Arg890His)	Exon 12	1	VUS-Warm	Wulczyn et al., 2019 (43)
	c.4645A>G	p.(Met1549Val)	Exon 25	5	LP	Scholl et al., 2015 (26)
	c.4647G>C	p.(Met1549Ile)		1	VUS-Hot	Daniil et al., 2016 (27)
	c.6248C>T	p.(Pro2083Leu)	Exon 35	1	VUS-Cold	
*CACNA1D*	c.1208G>A	p.(Gly403Asp)	Exon 8	1	LP	Scholl et al., 2013 (25)
	c.2310C>G	p.(Ile770Met)	Exon 17	1	LP	
	c.776T>A	p.(Val259Asp)	Exon 6	1	VUS-Warm	Semenova et al., 2018 (44)
	c.812T>A	p.(Leu271His)		1	VUS-Warm	De Mingo Alemany et al., 2020 (45)
**(early onset) Hypertension**						
*KCNJ5*	c.775G>A	p.(Val259Met)	Exon 2	1	VUS-Tepid	Markou et al., 2015 (46)
	c.834T>A	p.(His278Gln)		1	VUS-Tepid	Qin et al., 2019 (47)
	c.1042T>A	p.(Tyr348Asn)	Exon 3	1	VUS-Tepid	Markou et al., 2015 (46)
	c.1123C>T	p.(Arg375Trp)		1	LB	Qin et al., 2019 (47)

^1^ RefSeq reference transcript: NM_004366.6 (CLCN2)/NM_000890.5 (KCNJ5)/NM_021098.3 (CACNA1H)/NM_000720.4 (CACNA1D); ^2^ ACMG/AMP five-tier system: B (Benign), LB (Likely benign), P (Pathogenic), LP (Likely pathogenic), VUS (Variant of uncertain significance); ^3^ ACGS (Association for Clinical Genomic Science) VUS temperature scale: Ice Cold, Cold, Cool, Tepid, Warm, Hot.

In 2008, individuals with childhood-onset PA, resistant AH, hypokalemia and bilateral macronodular adrenal hyperplasia were reported (31). In 2011, an inactivating germline variant in the *KCNJ5* gene was identified in a case with a similar clinical presentation. Named FH 3 (OMIM #613677), this autosomal dominant PA subtype is caused by an impaired function of K^+^ GIRK4 (Kir3.4) potassium channel, which is encoded by *KCNJ5* gene (23).

The molecular defect in the K^+^ GIRK4 potassium channel leads to the loss of its ionic selectivity, with a consequent increase in sodium conductance (**Figure 1B**). Naturally responsible for maintaining the zona glomerulosa membrane potential, it starts to act as a channel in favor of sodium influx, promoting continuous membrane depolarization and subsequent activation of voltage-dependent Ca^+^ channels. These increased intracellular calcium concentrations trigger *CYP11B2* overexpression and aldosterone synthesis (39).

The genetic study of numerous PA cohorts and the consequent mapping of new *KCNJ5* pathogenic variants allowed, over the years, to expand the phenotypic heterogeneity of this PA subtype (39–41) (**Table 2**). Certain alleles between amino acids residues 151-158 of the K^+^ GIRK4 potassium channel, more specifically p.(Gly151Arg), p.(Ile157Ser), and p.(Thr158Ala), are correlated with a more severe PA clinical presentation, with early-onset hypertension, more resistant to drug treatment and with a frequent need for bilateral adrenalectomy (24). On the other hand, some substitutions in this same region, namely p.(Gly151Glu) and p.(Tyr152Cys), result in a mild clinical presentation, with an adequate blood pressure control with aldosterone antagonists and without evidence of adrenal hyperplasia in CT evaluation (13, 39). Interestingly, *in vitro* experiments showed that mutant *KCNJ5* channels can be undermined with the use of macrolide antibiotics such as roxithromycin and clarithromycin, suppressing *CYP11B2* expression and aldosterone production (49).

Four *KCNJ5* germline variants were reported in cohorts of patients with AH without a typical familial and biochemical diagnosis of PA (**Table 2**) (46, 47). The p.(His278Gln) variant, for example, was reported in a patient with resistant AH with normal serum K^+^ levels, plasma renin activity and aldosterone levels. The allele was inherited from his father who had essential AH without PA (47). None of the other reported cases had a phenotype similar to FH 3 patients, with early-onset medication-resistant hypertension, hypokalemia and bilateral adrenal macronodular hyperplasia (24).

Type 4 FH (OMIM #617027), the rarest subtype of PA, is caused by gain-of-function germline variants in *CACNA1H* gene (autosomal dominant inheritance), which encodes calcium voltage-gated channel subunit α1 H (Ca_v_3.2) (26) (**Tables 1**, **2**). Scholl et al. identified a recurrent heterozygous variant in the *CACNA1H* gene in five patients with early-onset PA (26). *In silico* studies with the identified p.(Met1549Val) mutant demonstrated an increase in calcium influx into zona glomerulosa cells, resulting in continual stimulation of aldosterone synthesis (50). Later studies demonstrated a late and incomplete penetrance of this PA subtype (27).

In 2013, Scholl et al. sequenced the candidate *CACNA1D* gene in 100 unrelated individuals with early-onset PA and identified two *de novo* heterozygous alleles in two girls with an undescribed syndrome featuring PA, AH, seizures and neuromuscular abnormalities (OMIM #615474) (25) (**Table 2**). This gene encodes the α 1D subunit of the L-type voltage-gated Ca^2+^ channel Ca_v_1.3. The identified variants promote an activation of the Ca^+2^ channel at lower depolarization potentials, resulting in increased Ca^+2^ influx (25). Subsequently, two more cases were reported with *de novo* heterozygous *CACNA1D* variants, leading to a severe developmental disorder also associated with developmental delay, intellectual impairment, neurological symptoms (including seizures), and endocrine symptoms, evident as PA and/or congenital hyperinsulinemic hypoglycemia (44, 45).

Recently, rare heterozygous missense germline variants in the phosphodiesterase 2A (*PDE2A*) and 3B (*PDE3B*) genes were identified in 3 out of 11 patients with PA caused by bilateral hyperplasia (51). In addition, PDE2A was a marker of zona glomerulosa and aldosterone-producing hyperplastic areas and micronodules. *In vitro* functional studies supported the involvement of PDE2A and PDE3B in the pathogenesis of bilateral PA. PKA activity in frozen tissue was significantly higher in adrenals from patients harboring *PDE2A* and *PDE3B* variants. Interestingly, inactivating *PDE2A* and *PDE3B* variants increased SGK1 and SCNN1G/ENaCg at mRNA or protein levels (51).

SGK1 (serum and glucocorticoid inducible kinase-isoform 1) belongs to a large family of serine-threonine kinases. SGK1 is expressed in numerous tissues and plays a major role in transmembrane ionic transport, being established as an important regulator of Na^+^ transporters (52). Aldosterone is the most notorious hormonal regulator of SGK1 expression. After binding to the cytosolic mineralocorticoid receptor, aldosterone promotes the transcription of SGK1, which regulates a variety of ion transporters, such as ENaC (epithelial sodium channel). SGK1 reduces ENaC ubiquitination and degradation, as well as its cellular internalization (53). Therefore, *PDE2A* and *PDE3B* variants can induce aldosterone signaling by increasing SGK1/SCNN1G(ENag) (51). In addition, an increase in SGK1 activity also stimulates hypercoagulability, fibrosis and inflammation processes (54).

## Unilateral Primary Aldosteronism

Aldosteronomas are a major cause of unilateral PA, associated with somatic variants in *KCNJ5*, *CACNA1D*, *ATP1A1*, *ATP2B3*, *CLCN2*, *CACNA1H* and *CTNNB1* genes (**Table 3**). These genes drive autonomous aldosterone production and/or directly contribute for tumorigenesis (68). In 2011, Choi et al. identified recurrent *KCNJ5* gain-of-function variants in aldosteronomas, namely p.(Gly151Arg) and p.(Leu168Arg), that affects residues at the channel ion selectivity filter (23) (**Table 4** and **Figure 1B**).

**Table 3:** Genetic causes of unilateral primary aldosteronism.

Gene (OMIM)	First Report	Prevalence	Discovery Approach	Features
*KCNJ5* (*600734)	2011 (23)	>40%	Cohort | Candidate Gene	Larger APAs with predominance of ZF-like clear cell composition; More frequent in younger, females, and East Asian patients; High aldosterone levels and severe hypokalemia (55–58)
*ATP1A1* (*182310)	2013 (59)	5.3%	Cohort	More frequent in male patients; APA with predominance of compact ZG-like cells, smaller size* (56, 60)
*ATP2B3* (*300014)	2013 (59)	1.7%	Cohort	APA with predominance of compact ZG-like cells; Severe hypokalemia (56, 60)
*CACNA1D* (*114206)	2013 (61)	9.3%	Cohort | Candidate Gene	More frequent in black and male patients; APA with predominance of compact ZG-like cells, smaller size* (56, 62)
*CTNNB1* (*116806)	2015 (63)	5%	Cohort | Candidate Gene	More frequent in female and older patients; Associated with pregnancy and menopause; Higher *LHCGR* and *GNRHR* gene expression (63, 64)
*CLCN2* (*600570)	2018 (28)	<1%	Cohort | Candidate Gene	Found in younger patients with high aldosterone levels; APA with smaller size** (65, 66)
*CACNA1H* (*607904)	2020 (67)	<1%	Cohort | Candidate Gene	Intra-tumoral *CYP11B2* expression heterogeneity; Composed of compact ZG-like cells** (67)

*Compared with KCNJ5 tumors; **Few (<3) cases reported in the literature, no statistical relevance; APA, aldosterone-producing adenomas (aldosteronomas); ZF, zona fasciculata; ZG, zona glomerulosa.

**Table 4:** Somatic variants identified in adrenal lesions associated with unilateral primary aldosteronism.

Gene	Nucleotide change^1^	Aminoacid change^1^	Region	Reference(first report)
*KCNJ5*	c.451G>A	p.(Gly151Arg)	Exon 2	Choi et al., 2011 (23)
	c.503T>G	p.(Leu168Arg)		
	c.433G>C	p.(Glu145Gln)		Akestrom et al., 2012 (69)
	c.472A>G	p.(Thr158Ala)		Mulatero et al., 2012 (38)
	c.451G>C	p.(Gly151Arg)		Taguchi et al., 2012 (70)
	c.467_469del	p.(Ile157del)		Azizan et al., 2012 (58)
	c.433G>A	p.(Glu145Lys)		Azizan et al., 2013 (61)
	c.446insAAC	p.(Thr149_Ile150insThr)		Kuppusami et al., 2014 (71)
	c.376T>C	p.(Trp126Arg)		Williams et al., 2014 (72)
	c.461T>G	p.(Phe154Cys)		Scholl et al., 2015 (73)
	c.470_471delinsAA	p.(Ile157Lys)		
	c.450_451insATG	p.(Ile150_Gly151insMet)		
	c.433_434insCCATTG	p.(Ile144_Glu145insAlaIle)		
	c.445_446insGAA	p.(Thr148_Thr149insArg)		Zheng et al., 2015 (74)
	c.439G>C and c.448_449insCAACAACCA	p.(Glu147Gln) and p.(Thr149_Ile150insThrThrThr)		Wang et al., 2015 (75)
	c.457_492dup	p.(Gly153_Gly164dup)		
	c.343C>T	p.(Arg115Trp)		Cheng et al., 2015 (76)
	c.737A>G	p.(Glu246Gly)		
	c.445A>T	p.(Thr149Ser)		Nanba et al., 2016 (77)
	c.443C>T	p.(Thr148Ile)		
	c.432_439delinsCA	p.(Glu145_Glu147delinsLys)		Zheng et al., 2017 (78)
	c.414_425dup	p.(Ala139_Phe142dup)		Hardege et al., 2015 (79)
	–	p.(Gly184Glu)*		Kitamoto et al., 2018 (80)
	–	p.(Ile157_Glu159del)*		
	–	p.(Gly151_Tyr152del)*		
	c.420C>G	p.(Phe140Leu)		Nanba et al., 2018 (81)
	c.447_448insATT	p.(Thr149delinsThrIle)		
	c.445_446insTGG	p.(Thr149delinsMetAla)		Nanba et al., 2019 (62)
*CACNA1D*	c.4007C>G	p.(Pro1336Arg)	Exon 32	Azizan et al., 2013 (61)
	c.4062G>A	p.(Met1354Ile)		
	c.2239T>C	p.(Phe747Leu)	Exon 16	
	c.2969G>A	p.(Arg990His)	Exon 23	
	c.776T>A	p.(Val259Asp)	Exon 6	
	c.2241C>G	p.(Phe747Leu)	Exon 16	
	c.2250C>G	p.(Ile750Met)		Scholl et al., 2013 (25)
	c.4012G>A	p.(Val1353Met)	Exon 33	
	c.2239T>G	p.(Phe747Val)	Exon 16	
	c.1207G>C	p.(Gly403Arg)	Exon 8A	
	c.1955C>T	p.(Ser652Leu)	Exon 14	Fernandes-Rosa et al., 2014 (56)
	c.2222A>G	p.(Tyr741Cys)	Exon 16	
	c.2993C>T	p.(Ala998Val)	Exon 23	
	c.3455T>A	p.(Ile1152Asn)	Exon 27	
	c.3451G>T	p.(Val1151Phe)		
	c.2936T>A	p.(Val979Asp)	Exon 23	
	c.1964T>C	p.(Leu655Pro)	Exon 14	
	c.2943G>C	p.(Val981Asn)	Exon 23	
	c.2248A>T	p.(Ile750Phe)	Exon 16	
	c.2992_2993delinsAT	p.(Ala998Ile)	Exon 23	
	c.2182G>A	p.(Val728Ile)	Exon 15	Wang et al., 2015 (75)
	c.2240T>G	p.(Phe747Cys)	Exon 16	Nanba et al., 2016 (82)
	c.3458T>G	p.(Val1153Gly)	Exon 27	Tan et al., 2017 (83)
	c.776T>G	p.(Val259Gly)	Exon 6	Nanba et al., 2018 (81)
	c.1201G>C	p.(Val401Leu)	Exon 8	Akerstrom et al., 2015 (84)
	c.1229C>T	p.(Ser410Leu)	Exon 9	Backman et al., 2019 (85)
	c.3019T>C	p.(Cys1007Arg)	Exon 24	Nanba et al., 2019 (62)
	c.3044T>G	p.(Ile1015Ser)		
	c.926T>C	p.(Val309Ala)	Exon 7	
	c.2978G>C	p.(Arg993Thr)	Exon 23	
	c.2968C>G	p.(Arg990Gly)		
	c.1856G>C	p.(Arg619Pro)	Exon 13	
	c.3452T>C	p.(Val1151Ala)	Exon 27	Guo et al., 2020 (86)
	c.2240T>C	p.(Phe747Ser)	Exon 16	
	c.2261A>G	p.(Asn754Ser)		
	c.2978G>T	p.(Arg993Met)	Exon 23	
	c.2906C>T	p.(Ser969Leu)	Exon 22	Nanba et al., 2020 (87)
	c.3044T>C	p.(Ile1015Thr)	Exon 24	De Sousa et al., 2020 (88)
*ATP1A1*	c.311T>G	p.(Leu104Arg)	Exon 4	Beuschlein et al., 2013 (59)
	c.299_313del	p.(Phe100_Leu104del)		
	c.995T>G	p.(Val332Gly)	Exon 8	
	c.2878_2887delinsT	p.(Glu960_Ala963delinsSer)	Exon 21	Azizan et al., 2013 (61)
	c.295G>A	p.(Gly99Arg)	Exon 4	Williams et al., 2014 (72)
	c.306_317del	p.(Met102_Trp105del)		Akerstrom et al., 2015 (84)
	c.304_309del	p.(Met102_Leu103del)		
	c.308_313del	p.(Leu103_Leu104del)		
	c.2867_2882delinsG	p.(Phe956_Glu961delinsTrp)	Exon 21	
	c.2877_2882del	p.(Phe959_Glu961delinsLeu)		
	c.2879_2890del	p.(Glu960_Leu964delinsVal)		
	c.2864_2878del	p.(Ile955_Glu960delinsLys)		Nanba et al., 2019 (62)
	c.2878_2892delinsGCCGTG	p.(Glu960_Leu964delinsAlaVal)		Nanba et al., 2018 (81)
	c.2874_2882del	p.(Phe959_Glu961del)		Guo et al., 2020 (86)
	c.2877_2888del	p.(Glu960_Ala963del)		
	c.2878_2895delinsGCCCTGGTT	p.(Glu960_Ala965delinsAlaLeuVal)		Nanba et al., 2020 (87)
*ATP2B3*	c.1272_1277del	p.(Leu425_Val426del)	Exon 8	Beuschlein et al., 2013 (59)
	c.1277_1282del	p.(Val426_Val427del)		
	c.1273_1278del	p.(Leu425_Val426del)		
	c.1270_1275del	p.(Val424_Leu425del)		Fernandes-Rosa et al., 2014 (56)
	c.1277_1298delinsACA	p.(Val426Aspfs*10)		Scholl et al., 2015 (73)
	c.1264_1278delinsAGCACACTC	p.(Val422_Val426delinsSerThrLeu)		Zheng et al., 2015 (74)
	c.1276_1287del	p.(Val426_Val429del)		Akerstrom et al., 2015 (84)
	c.1228T>G	p.(Tyr410Asp)		Wu et al., 2015 (89)
	c.1269_1274del	p.(Val424_Leu425del)		Murakami et al., 2015 (90)
	c.1279_1284del	p.(Val427_Ala428del)		Kitamoto et al., 2016 (60)
	c.1281_1286del	p.(Ala428_Val429del)		Dutta et al., 2014 (91)
	c.1264_1275delinsATCACT	p.(Val422_Leu425delinsIleThr)		Nanba et al., 2018 (81)
	c.367G>C	p.(Gly123Arg)	Exon 2	Backman et al., 2019 (85)
*CACNA1H*	c.4289T>C	p.(Ile1430Thr)	Exon 22	Nanba et al., 2020 (67)
*CLCN2*	c.71G>A	p.(Gly24Asp)	Exon 2	Dutta et al., 2019 (65)
	c.64-2_74del	p.(Met22fs)		Rege et al., 2020 (66)

^1^ RefSeq reference transcript: NM_000890.5 (KCNJ5)/NM_001128839.3 (CACNA1D)/NM_000701.8 (ATP1A1)/NM_001001344.2 (ATP2B3)/

NM_004366.6 (CLCN2)/NM_021098.3 (CACNA1H); * Nucleotide change not provided by authors.

*KCNJ5* is the most frequently affected gene in aldosteronomas (>40%), with even higher prevalence in Japanese and/or Eastern Asian cohorts (65-69% approximately). Characteristically, *KCNJ5* mutant aldosteronomas are more frequent in female (>70%) and younger patients, with larger tumor size. Higher preoperative aldosterone and reduced potassium levels were also identified in these patients, which could contribute to early-onset disease, severity and earlier diagnosis (23, 24, 55, 74, 92).

In 2013 after *KCNJ5* discovery, somatic *CACNA1D* gain-of-function variants were reported in aldosteronomas, with a prevalence of around 10%. *CACNA1D* encodes the α1 subunit Ca_v_1.3 of a voltage dependent L-type (long-lasting) calcium channel and its pathogenic variants affect conserved residues within the channel activation gate (**Table 3**). Compared to wild-type, mutated Ca_v_1.3 reaches activation in less depolarized membrane potentials, causing abnormal Ca^+^ influx, *CYP11B2* expression, and aldosterone production (**Table 4** and **Figure 1B**). In contrast with *KCNJ5* related aldosteronomas, *CACNA1D* tumors are significantly smaller and more frequent in older male patients (25, 56, 61).

In 2013, Beuschlein et al. identified somatic variants in genes encoding ATPases, *ATP1A1* and *ATP2B3* in aldosteronomas (59). Missense and in-frame deletion variants in *ATP1A1*, which encodes Na^+^/K^+^ ATPase α subunit, impair pump activity and significantly reduce affinity for potassium, resulting in inappropriate membrane depolarization (**Table 4** and **Figure 1B**). *ATP2B3* encodes a Ca^+^ ATPase in which loss-of-function alleles (in-frame deletions) lead to a loss of physiological pump function, responsible for sodium and possibly calcium ions leaking into the cell, inducing membrane depolarization, and contributing to increased calcium concentrations. The combined prevalence of somatic variants in ATPases is around 7% and, until now, no ATPase pathogenic variants were found as germline or surrounding aldosteronoma tissue. Additionally, ATPase mutant aldosteronomas showed a high prevalence among older male patients (61, 93).

As found in other adrenocortical tumors, somatic gain of function variants in *CTNNB1* gene, encoding β catenin, also have been reported in around 5% of aldosteronomas (**Tables 3**, **4**). Affected adrenals had an aberrant β catenin accumulation in the Wnt cell-differentiation pathway and overexpression of luteinizing hormone/choriogonadotropin receptor (LHCGR) and gonadotropin-releasing hormone receptor (GnRHR) (63, 94, 95). Patients harboring aldosteronomas with *CTNNB1* variants are more frequently females (60-70%) and older individuals, with no significant differences in preoperative aldosterone levels, tumor size and frequency familial hypertension compared with those with *KCNJ5* variants (64). Unfortunately, the underlying mechanism leading to *CYP11B2* overexpression due to *CTNNB1* mutations remains unclear. Berthon et al. (96) showed that β-catenin plays an essential role in the control of basal and Angi II-induced aldosterone secretion, by activating *AT1R*, *CYP21* and *CYP11B2* transcription (96).

Due to recent advances in high throughput sequencing, few somatic variants have been recently identified in 2 genes only so far related to FH (*CLCN2* and *CACNA1H*): the missense p.(Gly24Asp) (*CLCN2*), previously reported in FH 2 (28, 65); the splice junction loss c.64-2_74del (*CLCN2*) (65), and more recently, the missense p.(Ile1430Thr) (*CACNA1H*) (**Table 4**) (67).

The knowledge about adrenal lesions associated with PA and the detection rate of somatic variant have been significantly changed since the development of highly specific monoclonal antibodies against CYP11B1 and CYP11B2 (97). Under normal conditions, CYP11B2 was sporadically detected in the zona glomerulosa, whereas CYP11B1 was entirely detected in the zonae fasciculata-reticularis (98). In younger individuals, immunohistochemistry from normal adrenals reveals a continuous CYP11B2 expression throughout the ZG layer, but this pattern changes in adults and CYP11B2 expression becomes discontinuing in ZG (98, 99). Next, Nanba et al. demonstrated that CYP11B2 immunostaining was a powerful tool for histopathological identification of adrenal lesions associated with aldosterone overproduction (100).

Fernandes Rosa et al. performed the most comprehensive study in a cohort of 474 aldosteronomas from the European Network for the Study of Adrenal Tumors, reaching a detection rate of somatic variants of 54%, although *CTNNB1* sequencing was not included in this study (56). Two other studies, which included *CTNNB1* sequencing, demonstrated similar findings: Wu et al. studied 219 aldosteronomas, detecting somatic variants in 58.4% of them (101), and Vilela et al. reported a discovery rate of approximately 50% (102).

Recent studies using immunohistochemistry-guided approach to determine the exact source of abnormal aldosterone production led to the identification of pathogenic somatic variants in around 90% of screened aldosteronomas (81, 82, 88, 103). The lower prevalence of somatic variants found in aldosteronomas in previous studies using conventional approaches, not taking in account CYP11B2 expression, is explained due to the macroscopical selection of non-aldosterone-producing adrenal lesions (81). A recent review confirmed these previous findings, showing a higher detection rate of somatic variants with CYP11B2-guided extraction (85%) when compared to the classical approach with DNA extraction from fresh frozen tissue (54%) (57). Overall, the variant-negative ratio decreased from 46% to 15%. Gene-specific detection rate also increased from 34% to 56% in *KCNJ5*, 8% to 10% in *CACNA1D*, 8% to 12% in *ATP1A1* and 4% to 5% in *ATP2B3* (57).

Moreover, the CYP11B2-guided high throughput sequencing method has revealed a wide complexity of aldosterone-producing lesions in patients with PA (81, 82, 88, 103, 104). In multinodular cases, tumors from the same adrenal might harbor different recurrent somatic variants, suggesting independent triggers for the somatic events (82, 105). Interestingly, aldosterone production may occur from multiple sources: multiple aldosteronomas in the same adrenal gland, dominant non-producing adenoma with satellite CYP11B2 positive non-dominant nodules, and clusters of autonomous aldosterone-producing cells (APCCs) without apparent neoplasia (55, 81, 88, 106, 107).

APCCs are common in normal adrenals and accumulate with age, becoming more often detectable in morphologically normal adult adrenals (108). Somatic pathogenic variants in *CACNA1D, ATP1A1* and *ATP2B3* were found in 35% to 76% of the APCCs, with *CACNA1D* being the most mutated gene (108, 109). Interestingly, the spectrum of affected gene in APCCs is different from aldosteronomas. APCCs may a key player to the understanding of the physiology and pathophysiology of aldosterone production. It has been hypothesized that aldosteronomas can derive from APCCs with autonomous aldosterone production (harboring somatic in aldosterone-driver genes) (15, 99, 108).

Recently, the international histopathology consensus for unilateral PA (HISTALDO) classified the aldosterone-producing lesions (110). (**Table 5**). Aldosteronoma was defined as a well circumscribed CYP11B2-positive solitary neoplasm (≥ 10 mm diameter) composed of clear or compact eosinophilic cells or both cell types. Aldosterone-producing nodule is a CYP11B2-positive lesion (<10 mm diameter) morphologically visible with hematoxylin-eosin staining (“microaldosteronoma”). In this consensus, the nomenclature for APPC was changed to aldosterone-producing micronodules (APMs). APMs are defined as CYP11B2-positive lesion (<10 mm diameter) composed of ZG cells located beneath adrenal capsule. APMs are indistinguishable from normal zona glomerulosa (ZG) cells in hematoxylin-eosin staining (108, 110). In CYP11B2 staining, APMs have a strong uniform immunoreactivity for CYP11B2, without evident neoplasia or hyperplasia (108, 110).

**Table 5:** New histopathological nomenclature (HISTALDO) of aldosterone-producing adrenal lesions in patients with unilateral primary aldosteronism.

Aldosterone-producing Lesions (HISTALDO)	Size	HE visible	Histology
Aldosterone-producing adenoma (APA)	> 10mm	Yes	classical
Aldosterone-producing nodule (APN)	< 10mm	Yes	classical*
Aldosterone-producing micronodule (APM)	Microscopic	No	non-classical
Aldosterone-producing diffuse hyperplasia	Continuous layer of ZG cells	Yes	non-classical

*Non-classical in multinodular forms; HE, hematoxylin-eosin; ZG, zona glomerulosa.

These advances in PA histopathology allowed the definition of classical and non-classical histopathological features associated with PA (**Table 5**) (110). The classical histology is defined by the presence of a solitary aldosteronoma or APN. In contrast, “non-classical” histology is characterized by adrenals with multiple APNs or APMs *(*or multiple APMs and multiple APNs together) or aldosterone-producing diffuse hyperplasia (110, 111). In summary, non-classical histology is defined by the absence of a dominant aldosterone-producing lesion (such as a solitary aldosteronoma or APN). Interestingly, the mutational spectrum is different between classical and non-classical histology. *KCNJ5* somatic variants are predominant among aldosteronomas (classical histology), whereas *CACNA1D* is the most frequent mutated gene in APMs (81, 111).

## Impact on Clinical Outcome

The impact of genetic and clinical variables in outcome in PA patients have been more properly evaluated after the Primary Aldosteronism Surgical Outcome (PASO) study, which established criteria for clinical and biochemical success in unilateral PA patients after adrenalectomy (112). PASO criteria classified PA patients after adrenalectomy according to the biochemical outcome and clinical success. Complete biochemical success is defined by correction of hypokalemia when present pre-surgery and normalization of the aldosterone-to-renin ratio, and partial biochemical success as a correction of hypokalemia when present pre-surgery and a raised aldosterone-to-renin ratio, but with at least 50% decrease in baseline plasma aldosterone concentration compared to pre-surgical levels. Regarding blood pressure control, complete clinical success is defined as blood pressure <140x90 mmHg without anti-hypertensive medications after 6 months of follow-up, whereas partial clinical success as a reduction in the number or dose of anti-hypertensive medications when compared to pre-surgery (98).

Recently, non-classical histopathological lesions associated with aldosterone excess were found in 25% of the cases in a German cohort of unilateral PA (111). On the other side, APMs were found in only 5% (7 out of 137) of the cases in a Chinese cohort of unilateral PA (113). Therefore, additional studies from patients with different genetic backgrounds are essential to define the prevalence of classical and non-classical unilateral PA among different cohorts.

Of great importance in clinical practice, postsurgical complete biochemical success after adrenalectomy was correlated with histological features in a German cohort of unilateral PA. The rate of biochemical cure of PA was 98% in patients with the classical histopathology (solitary aldosteronoma or APN) compared with 67% in the patients with unilateral PA caused by non-classical histopathology (111). These findings suggested the presence of a baseline abnormal aldosterone production from the contralateral gland in patients with non-classical unilateral PA (**Table 5**).

*KCNJ5* somatic pathogenic variants have been associated with complete clinical success in cohorts of unilateral PA from Australia, West Norway, Japan and Brazil (80, 102, 114, 115). In a Brazilian cohort of PA, complete clinical success based in PASO criteria was more frequent in patients with aldosteronomas harboring *KCNJ5* pathogenic variants than in those with pathogenic variants in other driver genes (102). However, it should be emphasized that these previous studies did not conduct a genetic investigation based on CYP11B2 staining.

Interestingly, *KCNJ5* pathogenic variants have been more frequently detected in aldosteronomas (classical histopathology), which is associated with a higher chance of postsurgical complete biochemical success (57). Recently, somatic *KCNJ5* pathogenic variants were not associated with clinical and biochemical outcome in a small group of 38 aldosteronomas with genetic investigation guided by CYP11B2 staining. However, the influence of *KCNJ5* status in the outcome of PA patients cannot be ruled out and should be further evaluated in larger cohorts of unilateral PA with genetic investigation guided by CYP11B2 staining. Furthermore, the impact of somatic *KCNJ5* pathogenic variants on clinical outcome might depend on the frequency of classical histopathology among unilateral PA cases.

## Perspectives

Genetics of unilateral PA has remarkably improved in the last decade. However, most cases of bilateral hyperplasia remain without genetic etiology (15). Of great importance, a new histopathological classification has been recently proposed for aldosterone-producing lesions in unilateral PA (110). Besides the impact on the comprehension of PA pathophysiology, the histopathological features have influence in the outcome after unilateral adrenalectomy. *KCNJ5* mutational status and classical histopathology of unilateral PA (aldosteronoma) have emerged as relevant predictors of clinical and biochemical outcome, respectively (102, 111). Further studies will be important to characterize the spectrum of classical and non-classical unilateral PA among cohorts from different genetic backgrounds.

## Author Contributions

LS and AG participated on acquisition, analysis and interpretation of data, and drafted the manuscript. MA designed, drafted and revising critically the manuscript. All authors contributed to the article and approved the submitted version.

## Funding

This work was supported by Sao Paulo Research Foundation (FAPESP) grant 2019/15873-6 (to MA).

## Conflict of Interest

The authors declare that the research was conducted in the absence of any commercial or financial relationships that could be construed as a potential conflict of interest.

## Publisher’s Note

All claims expressed in this article are solely those of the authors and do not necessarily represent those of their affiliated organizations, or those of the publisher, the editors and the reviewers. Any product that may be evaluated in this article, or claim that may be made by its manufacturer, is not guaranteed or endorsed by the publisher.
